# High Prevalence of Sexually Transmitted Infections in Pregnant Women Living in Southern Brazil

**DOI:** 10.1097/OLQ.0000000000001276

**Published:** 2020-09-24

**Authors:** Nava Yeganeh, Regis Kreitchmann, Mei Leng, Karin Nielsen-Saines, Pamina M. Gorbach, Jeffrey Klausner

**Affiliations:** From the ∗ Department of Pediatrics, David Geffen School of Medicine at UCLA, Los Angeles, CA; † Irmandade da Santa Casa de Misericordia de Porto Alegre, Porto Alegre, Brazil; ‡ Federal University of Health Sciences of Porto Alegre; § UCLA Department of Medicine Biostats; ¶ Department of Epidemiology, Fielding School of Public Health at UCLA; ∥ Department of Internal Medicine, David Geffen School of Medicine at UCLA; ∗∗ Division of Infectious Disease, UCLA CARE Center, Los Angeles, CA

## Abstract

**Background:**

Porto Alegre, Brazil, has the highest rates of congenital syphilis and HIV in the country. Other treatable sexually transmitted infections (STIs) are associated with poor pregnancy and neonatal outcomes, but are only diagnosed by syndromic algorithms.

**Methods:**

Between September 2018 and November 2019, we offered all pregnant women clinic-based STI testing for HIV antibody and treponemal antibody (via lateral flow assay rapid tests provided by the Brazilian Government) and for *Neisseria gonorrhoeae*, *Chlamydia trachomatis*, and *Trichomonas vaginalis* (via polymerase chain reaction–based testing provided by Gene Xpert, Sunnyvale, CA) in 10 public prenatal health clinics in Porto Alegre. Participating women answered a brief survey via audio computer-assisted survey instrument regarding demographics, partnerships, and sexual behaviors. All infected individuals received appropriate treatment and referrals.

**Results:**

Of 400 pregnant women recruited, 94 (24%) were diagnosed with an STI, including 2% with HIV, 11% with syphilis, 9% with chlamydia, 1% with gonorrhea, 5% with trichomoniasis, and 3% with more than 1 STI. In our multivariate analysis, younger age (adjusted odds ratio [AOR], 1.1; 95% confidence interval [CI], 1–1.2), being non-White (AOR, 1.8; 95% CI, 1.1–3.1), having less education (AOR, 2; 95% CI, 1.2–3.4), and having a relationship <1 year (AOR, 2; 95% CI, 1.1–3.6) were all independent predictors of women having an STI. Endorsing symptoms of an STI (e.g., vaginal ulcers/lesions and vaginal discharge) was not predictive of having a laboratory-diagnosed STI (OR, 1.1; 95% CI, 0.7–1.7).

**Conclusions:**

Etiologic-based screening for STIs was uniformly accepted by women attending both hospital-based and primary health clinics in the south of Brazil and can result in appropriate treatment of pregnant women.

## Abstract 2

Almost 1 in 4 women being seen for prenatal care in our clinics in Porto Alegre Brazil were diagnosed with a sexually transmitted infection. Among women who tested positive, 59% did not have any symptoms.

Supplemental digital content is available in the text.

Women and men are at high risk of sexually transmitted infections (STIs) during pregnancy in Brazil, as most engage in condom-less vaginal and anal sex and up to 20% state they have multiple sexual partners.^1–4^ In addition, alterations in immune function^5^ and physiologic changes in the cervix during pregnancy may potentiate biological pathways of increased risk to infections, although this has not been seen with all STIs.^6,7^ Vulnerability to STIs during pregnancy is especially concerning because women may be asymptomatic, but untreated STIs can have severe health implications for their infants.

Acute HIV seroconversion among pregnant woman has well-documented adverse outcomes, including high maternal mortality and an increased risk of mother-to-child transmission of HIV.^8–14^ Both HIV-infected and HIV-exposed uninfected infants can face severe lifelong complications and high mortality rates.^15,16^ Similarly, other STIs during pregnancy are associated with adverse outcomes including stillbirth, low birth weight, infant blindness, and developmental delay and death. *Chlamydia trachomatis*, *Neisseria gonorrhoeae*, and *Trichomonas vaginalis* have all been associated with premature rupture of membranes, preterm birth, and low birth weight.^17–21^ Chlamydia and gonorrhea can cause neonatal conjunctivitis, and chlamydia is a cause of neonatal pneumonia.^22^ Syphilis can result in adverse pregnancy outcomes including stillbirth and preterm birth. In surviving infants, congenital syphilis affects the skeletal system, the lungs, and the brain with high morbidity and mortality.^23–26^ Appropriately diagnosing and treating STIs in women can contribute to improving maternal-fetal health and should remain a global priority.

Providers in Brazil routinely screen pregnant women for HIV and syphilis per the World Health Organization (WHO) guidelines,^27^ but in the south of Brazil, they do not have the infrastructure to actively perform etiologic screening for other curable STIs including chlamydia, gonorrhea, and trichomoniasis. Sexually transmitted infections other than HIV and syphilis are diagnosed based on symptoms or signs, despite the well-established poor sensitivity and specificity of syndromic management.^28,29^ Our study aims to assess the acceptability of offering STI testing and treatment to pregnant women in the south of Brazil, as well as evaluating the prevalence rates and risk factors for STIs.

## MATERIALS AND METHODS

From September 2018 to November 2019, we conducted a cross-sectional study of STIs in pregnant women by offering enrollment for STI etiologic screening to a convenience sample of 400 pregnant women seeking prenatal care at Santa Casa Hospital and at 10 primary clinics from the Public Health Department of Porto Alegre, Brazil. Enrollment was initiated in a hospital-based clinic, and then extended to primary health clinics in the surrounding communities after a pilot phase of 50 participants was completed. Of note, this study was designed to diagnose and treat pregnant women's male sexual partners in the prenatal setting. Data from male partners are still being analyzed and will be made available in a future study. For this reason, in order to participate, women had to be 18 years or older, be pregnant with plans to continue pregnancy, have a stable male partner for 3 months, and be able to provide informed consent. Women were excluded if they did not meet the inclusion criteria or reported a history of intimate partner violence. All women sitting in the waiting room were invited to participate in a study to improve the health of pregnant women. Potential participants expressing interest were invited to a private room to receive more information about the study and provide written informed consent when applicable.

### Protocol

After informed consent, women enrolled in the study received appropriate standard of care testing for HIV (BIOCLIN-HIV) antibodies, syphilis (Alere TP) treponemal antibody, hepatitis B antigen (VIKIA) testing, and hepatitis C (Alere HCV) antibody via finger-prick rapid test platform provided by the Brazilian Single Unified Health System, as well as immunizations for pertussis (Tdap), and influenza (if enrolled during the influenza season). As part of the study protocol, 2 vaginal swabs were collected from each participant, one for *C. trachomatis* and *N. gonorrhoeae* and one for *T. vaginalis* polymerase chain reaction (PCR) testing via the Gene Xpert platforms (Cepheid, Sunnyvale, CA). For patients scheduled to receive a vaginal examination during their prenatal care visit, usually in the primary care setting, providers collected 2 endocervical swabs per Gene Xpert instructions during the examination. For patients who were not receiving vaginal examinations, usually in the hospital-based setting, patients self-collected samples using provided collection swabs. Samples were placed in transport reagent and sent to the research office room where the Gene Xpert machine was located. Once office visits were completed, all samples were transferred to cartridges and testing was conducted. Of note, 4 of the results were indeterminate for *N. gonorrhoeae* and *C. trachomatis,* and 1 was indeterminate for *T. vaginalis*. Results were recorded and patients were notified of their results within 24 hours. Treatment was prescribed as described hereinafter.

While waiting for the results of their clinic-based rapid blood tests, each woman also answered a short survey via a handheld machine (audio computer-assisted survey instrument), asking her to describe her demographics, her relationship, her current pregnancy, past and current experiences with STIs, and sexual practices. Alcohol use and abuse were assessed using the AUDIT C 3-item alcohol screen.^30^

### Confirmation and Treatment

Women who had positive HIV rapid test results had a second confirmatory rapid test (BIOCLIN-HIV) performed. If both rapid test results were positive, the subject was classified as HIV infected in our analysis. Woman with any positive results for HIV was referred to our HIV specialists for evaluation. Each HIV-infected individual who was identified had standard-of-care HIV RNA viral load performed and was immediately referred to specific clinics for HIV-infected pregnant women for appropriate management including immediate treatment as per Brazilian guidelines. Women who had a point-of-care positive result for syphilis treponemal antibody received an injection of benzathine penicillin 2.4 million units intramuscularly × 1 according to Brazilian guidelines followed by referral to the laboratory for further evaluation via a Venereal Disease Research Laboratory (VDRL) titer. Women diagnosed with genital chlamydia infection received a prescription for azithromycin 1 g by mouth × 1. Women diagnosed with genital gonorrhea infection were given a prescription for ceftriaxone 500 mg by intramuscular injection. Women infected with trichomoniasis were given a single dose of metronidazole 2 g × 1 orally. All women were strongly encouraged to invite their respective partners to attend the clinic for STI testing as part of this study protocol. If their partner refused to come to the clinic for testing, partners were given a prescription or, in some cases, the treatment itself to deliver to their sexual partners via expedited partner therapy.

### Statistical Analysis

We stratified patients by site of prenatal care given that the hospital-based clinic serves as a referral center in Porto Alegre, Brazil, for more complicated pregnancies (advanced maternal age, preeclampsia, fetal anomalies) and may be less reflective of the general obstetric population seen in the primary health clinics. Two-sample *t* test and Pearson χ^2^ statistics were used to analyze continuous and categorical outcomes, respectively, and were used to describe this cohort's sociodemographic factors (age, race, sex, parity, schooling, employment, need for outside financial support), sexual risk factors (recent sexual activity including type of contact [oral, vaginal, receptive anal], age of sexual debut, partner type, number of partners, condom use), and other behavioral factors associated with STIs (alcohol and drug use during pregnancy). Univariate and multivariate logistic regression analyses were performed to assess predictors associated with any positive STI result, a positive syphilis rapid test result, a positive syphilis rapid test result with a VDRL titer >1:8, and a positive chlamydia PCR result. Of note, we also performed analysis using prevalence ratios and noted similar results; however, the confidence ratios were more narrow. All computations were done using SAS version 9.4. Institutional review board approval was obtained at the University of California, Los Angeles; Santa Casa Local Ethics Committee; Porto Alegre Health Municipal Department IRB; and the National Ethics Committee.

## RESULTS

Among 400 women recruited, 399 successfully completed the audio computer-assisted survey instrument interview. As shown in Table 1, 78 (20%) were recruited in hospital-based clinics, and the remaining were enrolled in affiliated primary health clinics. The mean age of enrolled women was 27 years. Most enrolled individuals (55.6%) self-identified as White, 20% self-identified as mixed, and 23.8% self-identified as Black. Seventy-eight percent were in a stable relationship lasting longer than a year, and 66% had completed elementary school. Notably, patients recruited at primary health clinics were less likely to be employed (47% in primary health vs. 64% in hospital based), more likely to be recruited earlier in pregnancy (recruited at gestational age of 23 weeks at primary health clinic vs. 31 weeks at hospital-based clinics), and less likely to have a planned pregnancy (45% in primary health clinics vs. 64% in hospital-based clinics). Among 94 women who had any STI, 55 (59%) did not report symptoms and would have not been appropriately diagnosed using syndromic management. Conversely, of the 161 women who described symptoms of increased vaginal discharge, pain with urinating, or genitourinary ulcers, only 29 (24%) were diagnosed with an STI using etiologic-based screening.

**TABLE 1:** Demographics and Behaviors of Pregnant Women in the Study, Stratified by Enrollment Site in Porto Alegre, Brazil

	All Women (n = 400)	Hospital-Based Clinics (n = 78)	Public Health Clinics (n = 321)	*P*
Age, median (range), y	26 (18–46)	28 (18–41)	25 (18–46)	0.15
Ethnicity, n (%)				0.607
White	222 (55.6)	46 (59)	176 (55)	
Black	95 (23.8)	15 (19)	80 (25)	
Mixed	78 (20)	17 (22)	61 (19)	
Length of relationship, n (%)				0.3
<1 y	89 (22)	14 (18)	75 (23)	
>1 y	310 (78)	64 (82)	246 (77)	
School, n (%)				0.137
Completed elementary	136 (34)	21 (27)	115 (36)	
>Elementary school	263 (66)	57 (73)	206 (64)	
Employed (yes), n (%)	203 (51)	50 (64)	153 (47.7)	0.009
Need to ask family for dollars in the last month (yes), n (%)	220 (55)	30 (38.5)	190 (59)	<0.001
First child, n (%)	229 (75)	55 (70.5)	244 (76)	0.34
Gestational age (range), wk	24.5 (4.6–41)	31.3 (7.1–41)	23 (4.6–40)	<0.001
Planned pregnancy, n (%)	193 (48)	50 (64)	143 (45)	0.002
Drink ETOH during pregnancy (yes), n (%)	109 (27)	15 (19)	94 (29)	0.076
Alcohol abuse (yes), n (%)	29 (7.3)	2 (2.6)	27 (8.4)	0.074
Drugs in past 6 mo, n (%)				0.02
Marijuana	30 (7.5)	2 (2.5)	28 (8.7)	0.06
Cocaine	10 (2.5)	0	10 (3)	0.06
Sex during pregnancy, n (%)				
Vaginal	342 (85.7)	70 (89.7)	272 (85)	0.5
Oral	222 (55.6)	46 (59)	176 (55)	0.7
Anal	86 (21.6)	17 (22)	69 (21.5)	1
Condom use (always), n (%)	38 (9.5)	7 (9)	31 (9.7)	0.65
Extramarital relationship (yes), n (%)	20 (5)	4 (5)	16 (4)	1
Fight in past week (yes), n (%)	227 (57)	31 (40)	196 (61)	<0.001
Has your partner been tested for HIV, n (%)	240 (60)	48 (62)	192 (60)	0.73
Do you know your partner's HIV results, n (%)	209 (52)	41 (53)	168 (52)	0.89
Same sex relationships, n (%)	3 (1)	0	3 (1)	1
Prior diagnosis with STI (yes), n (%)	94 (23.5)	11 (14.1)	83 (25.8)	0.03
STI symptoms (yes)	161 (40.3)	26 (33.3)	135 (42)	0.17

In the evaluation of potential risk factors for STIs, 27% of women admitted drinking alcohol during pregnancy, with 7.3% meeting the criteria for hazardous alcohol use. We performed an exploratory univariate analysis evaluating whether age, ethnicity (non-White), education, employment, and length of partnership or fighting with partner were associated with increased frequency of alcohol use, but did not find statistically or clinically significant associations. In the interview, 9.5% of women stated they used illicit drugs, with most self-reporting marijuana use. Most remained sexually active, with 86% of enrolled pregnant women reporting vaginal sex, 55.6% reporting oral sex, and 21.6% reporting anal sex. Only 9.5% stated they used condoms consistently during pregnancy, and 5% had extramarital relationships. Women recruited in primary health clinics were more likely to have argued with their sexual partner in the past weeks (61% vs. 31%) and endorsed more illicit drug use (9.5% vs. 2.6%).

**Figure 1:**
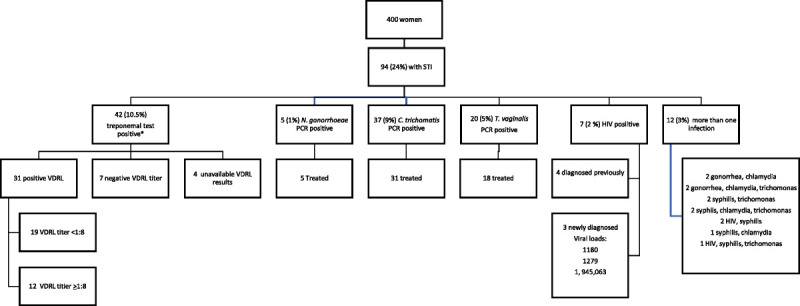
Flowchart of enrollment and distribution of STIs in pregnant women in the study. Among 400 women screened, 24% had a positive result for at least 1 STI, and 3% had positive results for more than 1.

All women accepted the STI testing offered; results are displayed in Figure 1. Forty-two (10.5%) had a positive treponemal antibody rapid test result, and of those, 31 (74%) had a positive confirmatory nontreponemal VDRL titer, with 12 having elevated VDRL titers >1:8. Treatment varied per provider. Some providers did not treat patients with low titer VDRL levels (<1:4) if there was a history of treatment of syphilis, but all women with a titer >1:8 did receive at least 1 dose of intramuscular benzathine penicillin. Thirty-seven women (9%) had a positive PCR for *C. trachomatis*, and 31 of those diagnosed (84%) were appropriately treatment. Failure to receive treatment was due to inability to contact woman after testing results were available despite 3 separate attempts. Twenty women (5%) had a positive PCR for *T. vaginalis*, and 18 (90%) were treated. Five (1%) women had a positive *N. gonorrhoeae* PCR, and all were appropriately treated. Twelve individuals (3%) had more than 1 STI diagnosed as listed in Figure 1. Of those diagnosed with HIV infection, 3 women were newly diagnosed during prenatal care. All individuals diagnosed with HIV infection had undetectable viral load levels by the time of delivery.

In our multivariate model shown in Table 2, younger age (adjusted odds ratio [AOR], 1.1; 95% confidence interval [CI], 1.03–1.2), non-White ethnicity (AOR, 2.1; 95% CI, 1.1–3.1), lower education (AOR, 2; 95% CI, 1.2–3.4), and shorter length of relationship (AOR, 2; 95% CI, 1.1–3.6) were each associated with having an STI. When evaluating syphilis and chlamydia separately, the risk factor profiles differed. Although non-White race (OR, 2.8; 95% CI, 1.4–5.5) and arguing with one's partner (OR, 2.3; 95% CI, 1.1–4.8) were risk factors for a positive syphilis rapid test result, these factors did not correlate with being infected with chlamydia. Similarly, younger age (OR, 1.3; 95% CI, 1.1–1.3), shorter length of relationships (OR, 2.3; 95% CI, 1.1–4.7), and outside sexual relationships (OR, 3.8; 95% CI, 1.2–11.3) were risk factors for chlamydia infection but not for syphilis. Multivariate analysis including race, age, education, recruitment site, alcohol use, drug use, outside sexual relationships, and condom use were performed for syphilis and chlamydia each, and revealed that non-White ethnicity was associated with a positive syphilis result (AOR, 2.5; 95% CI, 1.2–5.2) and having sexual relationships outside of the partnership was associated with being diagnosed with chlamydia (AOR, 4.5; 95% CI, 1.2–17.2). Examining frequency of chlamydia by age, we found that it affected 26 (16%) of 165 women between the ages of 18 and 24 years, 11 (5%) of 223 women between 25 and 40 years of age, and 0 of 11 women older than 40 years.

**TABLE 2:** Univariate and Multivariate Analyses of Factors Associated With Any STI, Syphilis, or Chlamydia Diagnosis in Pregnant Women

	Any STI		Syphilis Positive	Chlamydia Positive
	OR (95% CI)	AOR (95% CI)	OR (95% CI)	OR (95% CI)
Individual/Demographics				
Age*	0.9 (0.9–0.9)^†^	0.9 (0.9–1)^†^	0.9 (0.9–1)	0.8 (0.8–0.9)^†^
Ethnicity (non-White)	2.1 (1.3–3.4)^†^	1.8 (1.1–3.1)^†^	2.8 (1.4–5.5)^†^	1 (0.5–2.1)
Education (elementary vs. more than elementary)	2.4 (1.5–3.9)^†^	2 (1.2–3.4)^†^	1.5 (0.8–2.9)	1.7 (0.9–3.4)
Employed (no)	1.5 (1–2.5)		1.6 (0.8–3.1)	1.7 (0.9–3.5)
Asking for dollars from family (yes)	1.5 (0.9–2.4)		1.5 (0.8–3)	1.33 (0.7–2.7)
Unable to seek care due to money (yes)	1.2 (0.7–2)		1 (0.5–2)	1 (0.5–2.2)
Recruited at hospital-based clinic	0.5 (0.2–0.9)^†^	0.6 (0.3–1.3)	0.7 (0.3–1.6)	0.5 (0.2–1.4)
Gestational age at recruitment*	1 (0.99–1)		1 (0.99–1)	
Partnership				
Have other children (yes)	1.4 (0.8–2.5)		1.5 (0.7–3.3)	1.2 (0.5–2.8)
Planned pregnancy (yes)	0.6 (0.4–0.99)^†^		0.7 (0.4–1.3)	0.7 (0.4–1.4)
Length of relationship (<1 y)	2.6 (1.5–4.3)^†^	2 (1.1–3.6)^†^	1.5 (0.7–3)	2.3 (1.14–4.7)^†^
Fought with partner in the last week	2 (1.2–3.3)^†^		2.3 (1.1–4.8)^†^	1.9 (0.9–3.9)
Risk factors				
Drug use in 6 mo	1.8 (0.9–3.7)	1.1 (0.5–2.5)	2.1 (0.86–5.1)	1 (0.8–1.2)
Alcohol use during pregnancy	2.1 (1.3–3.5)^†^	1.7 (0.96–3)	1.4 (0.7–2.7)	1.9 (1–3.9)
Alcohol hazard score*	1.3 (1.1–1.5)^†^		1 (0.8–1.3)	1.3 (1.04–1.5)^†^
Age of sexual debut*	0.9 (0.8–1)		0.84 (0.7–1)	0.98 (0.8–1.2)
No. sex partners (<5 vs. > 20)	0.6 (0.2–1.8)		0.5 (0.12–1.7)	2.1 (0.3–16.5)
Vaginal sex during pregnancy	0.9 (0.5–1.8)		0.98 (0.4–2.4)	0.7 (0.3–1.6)
Anal sex during pregnancy	1.2 (0.7–2)		1.5 (0.74–3.1)	0.5 (0.2–1.4)
Oral sex during pregnancy	0.7 (0.4–1)		0.7 (0.4–1.3)	0.5 (0.3–1)
Condom use during pregnancy (yes)	1.8 (0.9–3.7)	1.9 (0.9–4.1)	1.4 (0.5–3.8)	1.6 (0.6–4.3)
Outside sexual relationships in pregnancy (yes)	2.3 (1.3–11.3)^†^	2.5 (0.9–7.4)	1.5 (0.4–5.5)	3.8 (1.2–11.3)^†^
Any STI symptoms/diagnoses?(yes)	1.1 (0.7–1.7)		1.4 (0.7–2.7)	1.3 (0.7–2.5)

*Age, gestational age, alcohol hazard score, and age of sexual debut were considered as continuous variables.

^†^*P* < 0.05.

## DISCUSSION

**Figure 2:**
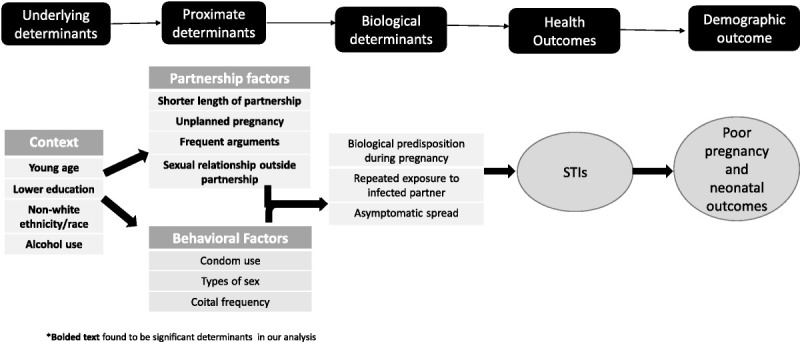
Proposed conceptual framework of factors increasing vulnerability to STIs during pregnancy.

Our study shows that almost 1 of every 4 women seeking prenatal care in our system's primary care clinics in Porto Alegre and its surrounding municipalities is infected with a treatable, if not curable, STI. Given that etiologic-based testing is not available in Porto Alegre for chlamydia, gonorrhea, and trichomoniasis, and symptoms did not correlate with risk of infection, more than half of the women diagnosed with these infections were unlikely to have been appropriately diagnosed or treated if these woman had not enrolled in our trial. Furthermore, 76% of women with symptoms did not have laboratory evidence of infection and would have inappropriately received antibiotic therapy using syndromic management. This study enrolled women older than 18 years who had stable partners for at least 3 months, so we may be underestimating the rate of STIs in pregnancy as we excluded younger women, those without a stable partner, and those with a concern for intimate partner violence. Despite this, our data suggest that the factors associated with having an STI during pregnancy include dynamics that may marginalize these women in society. Adapting the proximate determinates framework,^31s^Figure 2 shows the way underlying determinants such as demographic and social contexts (younger age, less education, non-White ethnicity, substance use) can inform proximate determinants including partnerships factors (shorter length of relationship, unplanned pregnancy, frequent arguments, outside sexual relationships) and behavioral factors (condom use, coital frequency) that can put women at increased risk of STIs and poor pregnancy outcomes.

The most common infection diagnosed was syphilis, which is unsurprising as Brazil has been experiencing a resurgence of syphilis and congenital syphilis, with the South being highly affected.^32s–35s^ To address this unfortunate trend, the Brazilian Unified Health System prioritized eliminating congenital syphilis since 2011^36s^ by offering rapid syphilis testing to all pregnant women in the first and third trimesters with treatment recommended with any positive results.^37s^ Despite these interventions, our study shows a prevalence rate of 10.5%. Of note, 7 (17%) of 42 likely represented old and/or treated infection and are unlikely to cause fetal harm, as confirmatory testing revealed negative VDRL titers. However, although it is difficult to appropriate stage syphilis based on laboratory work without a full physical examination and history, we noted that 12 (29%) of 42 had elevated titers >1:8, which could be consistent with acute or early infection, placing the infants at highest risk for adverse outcomes.^38s^ In our sample, all women with elevated titers greater than 1:4 received at least 1 dose of benzathine penicillin per WHO recommendation for early syphilis, and only 1 person received 3 doses of benzathine penicillin 1 week apart recommended for a diagnosis of syphilis with an unknown stage.^36s^ In general, given the high prevalence rate of syphilis in women with stable partnerships, treatment until VDRL results become available may be a reasonable approach. Our data agree with previously published reports in Brazil that non-White women seem to be at highest risk for syphilis.^26^^,33s^

Although the WHO has recommendations for screening pregnant women for HIV and syphilis, there are no specific recommendations for etiologic diagnosis of chlamydia or gonorrhea except syndromic management. Our results show that symptoms did not correlate with risk of positive PCR results for any STI, consistent with other studies showing that many STIs are asymptomatic in women.^29^^,39s^ Given that 16% of women between 18 and 24 years of age were diagnosed with chlamydia and the known association of this pathogen with preterm labor, low birth weight, perinatal mortality, HIV mother-to-child transmission, and neonatal conjunctivitis and pneumonia, laboratory-based screening should be considered, especially in younger women with shorter relationships or multiple partners.^21,25^^,40s,41s^

Finally, we noted high levels of alcohol use in up to 29% of women seeking care in primary health clinics; 8.4% of women met the AUDIT C screening criteria for hazardous drinking. This proportion is higher than cited in other studies in the south of Brazil evaluating alcohol use during pregnancy where only 9% to 13% of women endorsed any alcohol use during pregnancy.^2^^,42s^ This finding is highly concerning given the subtle, yet pervasive developmental abnormalities that can be associated with prenatal alcohol exposure.^43s^ We performed an exploratory analysis to examine risk factors associated with drinking, but no positive associations were found between demographics or partnership status/stability and alcohol use. Future studies should focus on ways to support women to abstain from alcohol in this period of time.

Our study was limited in that it only investigated the point prevalence of STIs and did not prospectively follow up women through pregnancy to investigate incident disease. There is a possibility that individuals may have received prior antibiotics for syndromic management of STIs, thus altering our disease prevalence or confounding our analysis. Furthermore, we were not able to retest all women for a “test of cure” or during the time of labor and delivery because of lack of testing kits and access to all women during the time of delivery. Despite these limitations, our study showed that etiologic-based screening for STIs was uniformly accepted by women attending both hospital-based and primary health clinics in the south of Brazil. Syphilis continues to be highly prevalent, and our study supports the current strategy of screening women multiple times during pregnancy. Symptoms of STIs did not correlate with having an STI, and etiologic-based laboratory screening should be considered, especially for younger women, those who endorse drinking during pregnancy, and those who are in unstable partnerships.

## Supplementary Material

SUPPLEMENTARY MATERIAL

## Appendix

For further references, please see “Supplemental References,” http://links.lww.com/OLQ/A548.
