# Supplementary material for: High Prevalence of Sexually Transmitted Infections in Pregnant Women Living in Southern Brazil
Source: Sex Transm Dis. 2020 Sep 24;48(2):128–33. doi: 10.1097/OLQ.0000000000001276 (PMC7817184; doi:10.1097/OLQ.0000000000001276)
Supplement: SUPPLEMENTARY MATERIAL [file olq-48-128-s001.docx]

31. Boerma JT, Weir SS. Integrating demographic and epidemiological approaches to research on HIV/AIDS: the proximate-determinants framework. The Journal of infectious diseases. 2005;191 Suppl 1:S61-67.

32. Domingues RM, Szwarcwald CL, Souza PR, Jr., et al. Prenatal testing and prevalence of HIV infection during pregnancy: data from the "Birth in Brazil" study, a national hospital-based study. BMC Infect Dis. 2015;15:100.

33. Serafim AS, Moretti GP, Serafim GS, et al. Incidence of congenital syphilis in the South Region of Brazil. Rev Soc Bras Med Trop. 2014;47(2):170-178.

34. Acosta LMW. Monitorando as metas do Comite da TV do HIC e SC de Porto Alegre. Secretaria Municipal de Saude de Porto Alegre—SMS 2018.

35. Ministerio da Saude. Boletim Epidemiologico de Sifilis- 2016. In Saude SdVe, (Ed). Brasilia, Brazil 2016.

36. World Health Organization. Eliminating Congenital Syphilis. In Department of Reproductive Health and Research WHO, (Ed). Geneva 2005.

37. Brazilian Ministerio da Saude. Institui, no ambito de Sistema Unico de Saude-a Rede Cegonha. . Brasilia: Diario Oficial da Republica Dederativa do Brazil 2011:Secao I, p. 109.

38. Center for Disease Control and Prevention. Sexually Transmitted Diseases Treatment Guidelines, 2015 MMWR. 2015;64(3).

39. Mudau M, Peters RP, De Vos L, et al. High prevalence of asymptomatic sexually transmitted infections among human immunodeficiency virus-infected pregnant women in a low-income South African community. Int J STD AIDS. 2018;29(4):324-333.

40. Goldenberg RL, Culhane JF, Iams JD, et al. Epidemiology and causes of preterm birth. Lancet. 2008;371(9606):75-84.

41. Adachi K, Nielsen-Saines K, Klausner JD. Chlamydia trachomatis Infection in Pregnancy: The Global Challenge of Preventing Adverse Pregnancy and Infant Outcomes in Sub-Saharan Africa and Asia. Biomed Res Int. 2016;2016:9315757.

42. Meucci RD, Saavedra JS, Saes da Silva E, et al. Alcohol intake during pregnancy among parturients in southern Brazil. Rev Bras Saude Mater Infant. 2017;14(4).

43. Williams JF, Smith VC, Committee On Substance A. Fetal Alcohol Spectrum Disorders. Pediatrics. 2015;136(5):e1395-1406
